# Hazards Faced by Young Designated Drivers: In-Car Risks of Driving Drunken Passengers

**DOI:** 10.3390/ijerph6061760

**Published:** 2009-06-08

**Authors:** Peter J. Rothe, Linda J. Carroll

**Affiliations:** 1 Centre for Health Promotion Studies and Alberta Centre for Injury Control and Research, School of Public Health, University of Alberta; 4075 RTF, 8308 – 114 Street; Edmonton, Alberta, Canada; T6G 2E1; 2 Department of Public Health Sciences and Alberta Centre for Injury Control and Research, School of Public Health, University of Alberta; 4075 RTF, 8308 – 114 Street; Edmonton, Alberta, Canada; T6G 2E1; E-Mail: lcarroll@ualberta.ca

**Keywords:** impaired driving, designated driver, traffic safety, injury control

## Abstract

This qualitative study explored the risk in the practice of young designated drivers transporting drunken peers. Young drivers 18–29 years old in Alberta, Canada participated in 12 focus groups (N = 146). Interviews were semi-structured. A key finding is that when highly intoxicated youth are driven by a designated driver who is a peer, they are likely to behave in ways that are unsafe. Unsafe actions of drunken passengers in the vehicle include physical “rough-housing” with the driver, creating stress for the driver that leads to high risk driving situations and disrupting safe driving through nausea and in-car vomiting.

## 1. Introduction

Impaired driving continues to be a high priority issue in injury prevention. A recent survey concluded that impaired driving (due to alcohol or illicit drugs) tops the list of the road safety concerns [1]. Recent data from Alberta, Canada gives substance to these concerns. Of drivers involved in injury collisions in 2007, 27% had consumed alcohol before the crash [2]. In 2006, nearly one-third of all traffic-related deaths in the United States involved alcohol [3]. The World Health Organization estimates that in European countries, alcohol is responsible for 45 percent of the burden of disability arising from motor vehicle crashes for men and for 18 percent of the burden for women [4].

Maximum blood alcohol concentrations (BAC) permitted for drivers vary widely across jurisdictions. For example, Romania and the Slovak Republic have zero-tolerance (that is, no BAC above zero is permitted), whereas a number of other jurisdictions (such as the United States and the United Kingdom) have set an upper BAC limit of 0.08 to 0.10 [5]. Penalties for impaired driving also vary widely, with most imposing increasing penalties for increasing BAC levels. For example, in most Canadian provinces, driving with a BAC of 0.05 or above is not permitted (through use of such strategies as temporary license suspension), while those drivers with a BAC of 0.08 or above are prosecuted under the Federal Criminal Code [6]. Minimum penalties under the Canadian Federal Criminal Code (for impaired driving) generally involve a substantial fine and a driving suspension, although penalties increase considerably if the impaired driver is involved in a crash, and penalties are even greater if someone is injured in a crash. Similar types of penalty are common across countries, although some jurisdictions (for example, California and several other states in the United States) impose jail time for first offenders even if there have been no crashes or injuries [7].

Impaired driving is an especially significant problem in young drivers [8]. Those aged 18 to 21 are more likely to have consumed alcohol prior to casualty collisions than any other age group [2,3]. Government agencies, community leaders and volunteer groups have developed, implemented and/or endorsed different kinds of projects and innovative strategies in attempts to reduce impaired driving-related injuries amongst young drivers. One common initiative is the designated driver program, currently popularized in Canada, the United States, and Europe. In Europe, this initiative is called the “European Bob” campaign, and it has become an instrumental component of sixteen European countries’ integrated approach to reduce drinking and driving. Australia also has endorsed the designated driver initiative by implementing a program called “Pick a Skipper”.

A designated driver is intended to be an individual with a driver’s license who is selected before celebrations to provide a safe and sober ride home to the others in the group. In order to ensure safety, the person identified as the designated driver is meant either to abstain from alcohol or, in accordance with the idea of harm reduction, to maintain a BAC level that is under the legal limit [9]. In this program, the onus is on the driver to be sober and the expectation is that passengers will reduce their frequency of riding with impaired drivers. Naturally, the promotion strategies and implementation of the designated driver program varies from country-to-country to reflect local situations and cultures.

Unfortunately, there is little evidence that the designated driver strategy has had much success in decreasing rates of impaired driving. In Australia, despite a 13% increase in respondents stating that they “always” select a designated driver, there was no actual change in self-reported alcohol-impaired driving or in self-reported riding with an alcohol-impaired driver [10]. In Europe, there has been little evaluation of designated driver programs. However, a recent Eurocare Report suggests that the limited information available on the effectiveness of designated driver programs suggests that these programs are less effective in preventing alcohol-impaired driving than originally envisioned and the report concludes that there is little evidence to date that designated driver programs lead to a reduction in drinking and driving [5].

In addition, the designated driver strategy, as it is currently practiced, suffers from a number of weaknesses. Of note is the fact that the “designated driver” may simply be the person in the group who is least drunk [8,9,11]. National telephone surveys of college students and young people in the United States suggest that only about half of designated drivers remain completely abstinent [9,12]. Another issue that has not been fully explored in the literature is the increased in-car risk created by drunken passengers when even sober designated drivers attempt to operate the vehicle. In a recent poll, 65% of those who had served as designated drivers reported feeling stressed in the car because they had to take care of sick or unconscious friends or control unruly behavior [15]. In that study, the authors contended that the effects of passengers on drivers generally, and the effect of drunken passengers on designated drivers specifically, need to be more fully explored to better judge the merits of designated driver programs. In fact, the designated driver strategy may actually lead to the driver’s companions increasing their alcohol consumption [13,14], which increases the difficulties faced by the driver.

Designated driving should be viewed as occurring within the larger interactional framework of peer relationships among the passengers and the designated driver, and should be examined within the context of the social, safety and legal systems that are embedded in the “taken-for-granted” normative rules, rituals, routines, etiquette and protocol practices of motor vehicle use. Within the physical confines of the vehicle might be a group of drunk and rowdy passengers. The designated driver is expected to “take-charge” as the driver and to be responsible for his or her drunken passengers; yet the driver is also still a peer, and may not have the ability (or the will) to control these passengers. The result is potential driving risk.

We present a qualitative study that explores the problematic nature of drunken passengers and designated drivers interacting in the vehicle. Briefly stated, we will outline the particularities of the in-car relationships and interactions that contribute to dangerous driving and potential crashes. Three dominant assumptions serve as scaffolds for this research. These assumptions arise from Goffman’s work in which he promotes the concept of “theatre” as a metaphor to describe social interactions. In Goffman’s framework, the social arena is the “theatre stage”, and the “theatrical performance” involves – in part – taking the roles that others expect of us, with the assumption of different roles in different situations. According to Goffman, when that performance is “disrupted” (when the roles are not carried out as expected), there is social disruption, with personal and interpersonal consequences [16]. The first of the three assumptions in the current study is that the motor vehicle’s interior represents a particular social arena, with the designated driver and drunken passengers interacting according to their perceived roles. The second assumption is that one of the important roles of the person assuming the task of designated driver (that is, the role involved in assuming the legal and ethical responsibility to maintain full control of the vehicle, regardless of the passengers’ behavior) is a relatively temporary one. Thirdly, in most cases the individual who assumes the temporary role as designated driver also has a more enduring role as peer and friend of their drunken passengers. Herein lays the crux of the issue. How can young designated drivers, who are transporting drunken passengers, behave in ways that maintain lawful and safe driving (the temporary role of “designated driver”) while still accommodating the peer relationship (the more enduring role)? The current study explores the difficulties faced by young designated drivers.

As a designated driver in the current study said:

Personally, I think it is hard to drive - to be the driver. You feel kind of left out cause everybody is having a blast and just you feel like you are kind of outside and that you should be right in there with them.

## 2. Methods

Our data come from 12 focus groups with young drivers. Focus group methodology has been shown to be useful in previous alcohol research studies [17,18]. The focus groups in this study were designed to: (a) explore the specific circumstances influencing designated driver behavior within the interactional context of vehicle, and to (b) delve into the normative practices and values of young designated drivers.

### 2.1. Participant Recruitment and Study Setting

Study participants were 146 Alberta residents, aged 18–29 years, who were identified and recruited with the assistance of community contacts. These community contacts were chosen because of their substantial knowledge of the inhabitants who lived in the area, and were local injury prevention coordinators, community health nurses and health promotion workers. Recruitment occurred in 12 different settings, selected to represent a mix of rural, small urban and large urban settings across the province of Alberta, Canada. The community contacts communicated with their local media outlets about the study, contacted regional employers and post secondary institutions for suitable study participants, explained the study to different organized groups in the community and publicized the study on relevant Internet sites.

### 2.2. Focus Groups

There were 12 focus groups, one in each study location, each lead by a trained and experienced qualitative research assistant. The interview format of the focus group was semi-structured and the size of the groups ranged from six to 12 participants.

The interview protocol was formulated around topic areas rather than specific questions; the topic areas and sample questions for the topic areas addressed in this study are listed in Table 1. The key objective for the focus groups was to gain a sense of the overall behavioral patterns of behavior of drunken passengers and designated drivers, and to understand the reasoning behind these behaviors.

### 2.3. Data Verification

We used a two-step data-verification process to help assure accurate data and relevant interpretation. The first step in the verification process for each focus group included consistent use of tagged responses, where the interview facilitator introduced such phrases as, “Do you mean that…,” “Are you saying that…” and “Give me an example of…” to ensure accurate understanding of what was being said and to attain a more embedded sense of meaning [19].

Secondly, inter-respondent verification procedures were used whereby respondents were asked about critical issues or anomalies that had been mentioned in earlier focus groups. For example, one of the key findings was that it was common for designated drivers to have consumed alcohol. However, in one of the early focus groups a respondent said that when he is chosen to be a designated driver he sometimes “sips” drink and at other times he “has a drink” of alcohol. When this question was posed in later interviews, it became clear that other participants also distinguished between “sipping” and “drinking” while serving as designated drivers.

### 2.4. Qualitative Interview Analysis

After obtaining informed consent, the interviews were recorded and transcribed verbatim. To ensure data accuracy, random monitoring (comparison of tape to transcription) was carried out. All 12 focus group interviews were treated as a single data set and the search for themes or patterns was conducted across the entire data set rather than within individual focus groups [19,20]. We considered a “theme” to be defined not only as an idea that occurred a number of times in the data set, but also as a verbalized form of reasoning that captured something important in relation to the overall research question, for example how illegal acts were normalized. These patterns were then pieced together to form themes, which were further synthesized to form a comprehensive picture of the participants’ collective experience.

## 3. Results

As a general finding, our focus groups highlighted the idea that the inside of a motor vehicle is a dynamic arena for drunken passengers to engage in high risk behaviors with little consideration of that risk. Analysis of these focus groups yielded several consistent findings.

### 3.1. In-Car Pranks

The combination of a group of youths and excessive drinking is frequently a recipe for rowdiness: “pranks” and jokes are a standard “lived experience” in these situations. The young people in our focus groups variously described “pranks” as “good fun”, “having a few laughs”, “teasing”, “just messing about” and “being there for a good time”. Focus group members described their drunken passengers as engaging in practical jokes that were considered funny, harmless, enjoyable and/or interesting from the drunken passengers’ perspective. However, from the designated driver’s perspective, these events were perceived as disruptive and often distressing. Although the pranks described did not typically involve anger, aggression or fighting, they were, nonetheless, a form of high risk-taking behavior that included a show of bravado before friends. The large amount of alcohol consumed by these passengers lead to a distortion of their judgments as to the limit of a good time or jokes that “go too far”. As retold by the following three designated drivers, young passengers are inclined to show off, be carefree foolish or reckless with others:

They (drunken passengers) all want to go somewhere different and they’re hanging out the open windows and yelling at people…. That’s pretty typical of for a bunch of drunks… (Female, Aged 25)

They just drink it (bottle of beer) before I get my hands on it. I reach back sometimes and that’s kind of dangerous on the road at night. So I pull over and they chug it and throw the bottle out the window. Sometimes they play “catch me” with an empty bottle while I’m driving. (Male, Aged 22)

They go out of their way to have fun in my vehicle and they do what they can to piss you off. (Female, Aged 21)

Some passengers were reported to have physically engaged the designated driver by hitting or choking them “for sport”, thereby endangering everyone in the car. There were reports of passengers grabbing the steering wheel, holding onto the transmission lever, tugging at the emergency brake or pulling at the driver’s seatbelt:

Yeah, we choke him out while he's driving. It’s all fun and games. It makes for a more interesting drive. (Male, Aged 19)

I think it’s hard, like they like to grab the steering wheel and steer for me and see it as funny…(Female, Aged 20)

I drove five people home in a three-person truck. There were two people in the drivers’ seat and one is laying across saying I will shift for you. So he grabs the transmission… (Female, Aged 20)

It is hard - people are pulling on your seat belt and saying “no I want to go here and go to another bar….” (Female, Aged 24)

All of these drunken passenger initiated pranks were spontaneous activities, considered to be part of “having fun,” with no aggressive purpose or intent to put anyone at risk. If not checked by the designated driver, the pranks could have resulted in a serious crash. It was therefore incumbent on the designated driver to stabilize the situation and control the actions of drunken passengers. But doing so may be seen as being of bad faith, a friend sanctioning other friends for having fun. As a young man noted about his group’s designated driver taking action against pranks:

She’s okay one moment then becomes a real drag. I don’t think we should drive with her no more. We want to just have fun and not be told what to do. (Male, Aged 20)

### 3.2. From Pranks to Disorderly Behavior

The intent of a “prank” is “good-natured fun”, despite the fact that the actual consequence may be more serious and the designated driver may be distressed by these “pranks”. However, “disorderly behavior” has more serious implications of hindrance, incapacitation and risk. Drunken passengers start pushing, shoving and fighting amongst each other, which is a flaring point for danger within the vehicle and potential crash. This may result in the designated driver feeling angry and fearful – some drivers report feeling incapable of taking action, and helpless in the situation. During the focus groups, this fear was generally expressed as feeling “unsafe”.

They get really rowdy, they want to wrestle in the car, it is hard to shoulder check, it is too loud they’re yelling… It is probably not safe at all cause they would bump me and it is just really not safe … (Female, Aged 22)

What do you expect from a…bunch of guys pissed to the eyeballs? They’re just crazy drunk, like they shoot elastics at each other. Then someone yells “stop it hurts,” and he jumps a guy to stop it and they shove and wrestle…. And I just do my thing with these crazy asses. I stay low. (Male, Aged 20)

I’ve been in tears driving them. They’re pushing and slapping each other in the back and then their stupid antics like grabbing someone’s cell phone and tossing it around like keep-away. It just leads to fights. And you know its coming, you just know…. I don’t drink so I put up with it. But it’s scary (Female, Aged 27)

According to our focus group participants, disputes amongst drunken passengers often started with innocent incidents like not sharing a bottle of beer, grabbing each other’s cell phones, arguing where to go for something to eat, shooting elastic bands at each other or insulting gestures. The inebriated state of some (especially male) passengers creates an upsurge of anger and shortens the distance between annoyance and physical retaliation. Play fights in the car become serious:

Well, being a designated driver myself for different events that, ah -, it’s no fun to try and get these drunk wandering people into a vehicle and then you get some guys swinging at each other… (Male, Aged 28)

I’m not a prude and I’m not going to say I’ve never gotten drunk in my life, but I’ve never started fighting in the car or feel all powerful because I’m drunk. Some guys are just like that. And they push the designated driver around. (Male, Aged 25)

My brother doesn’t drink…but when they all go out and drink he has to always drive someone home… He almost got into a serious accident because someone whacked him in the face. Some stupid drunk guy beside him hit him! (Female, Aged 22)

The designated driver’s responses are mixed, but a clear majority believes that they should take action, despite negative consequences to themselves. Responses ranged from passive icy stares to stern rejoinders, yelling and stopping the car until order prevailed:

My friends have actually…its come to the point where I would stay quiet and then say: “Fine go kill yourself. I ain't fighting you.” And that’s where it stops. They respect me for speaking up over their crazy ride. And it always works. (Female, Aged 27)

…When that happens I give them that look, the look that tells them be afraid, be very afraid (Male, Aged 23)

When fighting happens, I just stop the car and tell everybody to get out. My girlfriends sometimes get in the middle of the melee and I scream at them to stop… (Female, Aged 18)

Getting attacked, punched in the face! I don’t need that so, you know, I shut it down…. (Male, Aged 29)

Sometimes another guy will break up a fight between two guys, and sometimes a girl will get in between. Still, it pisses me off to no end…If they don’t stop when I yell at them then I just stop the car and get out and really yell at them…(Female, Aged 25)

However, others are unlikely to take action to deal with the social malaise in their vehicles. They do not want conflict and therefore stay passive or “grin and bear it” during drive, believing that action is futile. The following extracts demonstrate a view that was commonly shared in the focus groups:

I have a small car and they always pack in one on top of the other and they get in your face pretty well all of the time. They do everything to piss you off. I just grin and bear it (Female, Aged 23)

It’s hard. There’s loud people and all the windows are down and everybody is yelling…But there’s no need for me to get angry and yell back. It won’t do any good… (Female, Aged 22)

The boundary between the inside and outside of a car is fluid. Drunken passengers sitting in a vehicle can feel “anonymous” when taking action against other road users, who can observe or hear them. One such behavior is yelling obnoxious remarks to pedestrians. Consider the following:

I have a group of friends who like to, after the bar is closed, drive up and down (the avenue is named) yelling out the windows at people. And then the driver has to make sure not to hit any red lights and stuff like that. We gotta get out of there (Female, Aged 25)

Rather than attend to driving, the driver must deal with the drunken passenger-pedestrian outbursts and escape the situation as quickly as possible lest a retaliatory response is forthcoming from the offended party. Again, safety can be compromised.

### 3.3. Extending the Bar Behavior to the Vehicle

The bar or party is a locus of “good times” as exemplified through ample alcohol consumption. On occasion, the vehicle becomes an extension of the bar or party whereby earlier drunken behavior is transferred to the vehicle:

Some people are obnoxious when they get out of the bar and they don’t sit still and they bounce around and it all depends what happened before they left the bar. (Male, Aged 28)

The drunken passengers act in the moment, engaging in unlawful behaviors. More specifically, drunken passengers take alcohol into the car, or they open bottles of beer that they had hidden their purses or coats or that they had stashed under a seat. They may not actually drink the alcohol but use the glasses or bottles in dangerous ways. The situation is problematic for the designated driver:

My friends are kind of bastards, bringing beer into my car and waving it around where cops can see… (Female, Aged 21)

Yeah they scream at each other at the top of their voice and they spill beer on each other, like they’re having fun and they don’t give a damn what happens…(Female, Aged 19)

Two weeks ago we were at the bar and my friend took a glass of rum and coke in my car and threw it – like it hit my windshield and there was glass - and that kind of shit really annoys you but at least I’m not dead. (Male, Aged 21)

A bottle of beer may be passed amongst passengers, each one taking a sip before the empty bottle is thrown about in the car, tossed out the window or dropped on the floor. The designated driver may speak out or stop the vehicle in disgust. However, that response does little good because the beer has already been drunk and the action was already taken. A prevailing view is that as long as the driver doesn’t drink, why does it matter if the passengers “sip a few drinks” while en route? Another common attitude expressed is that passengers are allowed to drink in limos and on party busses (with a hired chauffeur), so why not in vehicles with designated drivers?

It is not uncommon for designated drivers to describe their drunken passengers as behaving like little children that require babysitting. They invoke a form of social accountability, the kind with which a parent could easily identify. Drunken passengers distract the driver and they stretch his/her patience and tolerance:

It’s definitely distracting…. To be the designated driver, you’re babysitting. You’ve got five kids in the car and it’s definitely distracting and frustrating. (Female)

Drunk people act like children right, like sometimes worse than children, they’re just obnoxious. They do dumb things, and they’re loud and so it’s very hard to drive with drunk people. I think it’s just hard dealing with them, listening to their drunken talk, nonsense… It’s loud it’s distracting. (Male)

Yea it’s hard, uh, it just bothers you, they’re like a bunch of wild kids and you just want them to shut up or whatever (Female)

Some interviewees claimed that drunken passenger continue to throw the kinds of objects (paper, Kleenex, empty cigarette boxes, glasses, other) that they had been throwing at the party they had just left. Whereas at the drinking event, they aimed at other guests or friends, in the vehicle they may aim for the designated driver. Discussion of “flying missiles” in the vehicle sometimes consumed extraordinary emphasis in focus group discussions. Several abbreviated comments illustrate the theme:

They are throwing stuff at your head and hanging out the window. It’s really very dangerous… (Female, Aged 26)

They act like jerks when we’re out and then it’s like that in my car when they, you know, throw crap at me (Male, Aged 22)

### 3.4. The Aftermath of Alcohol Abuse

There were those in the focus groups who strongly believed that designated drivers must have the courage to confront the worst of all in-car disturbances – vomiting. The issue was uniformly voiced as a crisis and condemned accordingly as devoid of bare minimum dignity. Opening a window was seen as the best response. There was a “code” expressed that a drunken passenger about to become nauseous must be near the open window, and that the designated driver should stop at the side of the road immediately to allow the passenger the opportunity to “get sick”. Of course, this move can be problematic if the car is in traffic and driving on the inside lane.

I remember having to clean puke off the side of my truck cause there wasn’t time to pull over and it was in the middle of winter. So by the time I got anywhere to deal with it, it was all hard. (Male, Aged 23)

If I’m like the designated driver and if they’re looking sick I’ll say keep the window down and if you are absolutely sick we’re stopping then I’ll stop on the side of the road like right now… (Male, Aged 21)

Sometimes if they are puking in the back you are thinking „don’t get it on the seat’ cause they have a garbage bag or whatever… (Female, Aged 21)

It appears that a fear of passengers vomiting is omnipresent and woven into the texture of drunken passenger in-car behavior. Some of the participants who served as designated drivers were vociferous about displaying their dislike for passengers vomiting in their vehicles:

Sometimes I get annoyed being the designated driver. I am like „ah don’t puke in the car, don’t touch the seat’. They are all having fun and it’s not fun for you really, like I dread it actually… (Female, Aged 27)

And then there’s the chance that someone is going to puke and you have to watch out for them while paying attention to your driving. Yeah it’s annoying. You just want to get home and you’re the last one to get to bed…It’s frustrating (Female, Aged 26)

Persistent passenger vomiting erodes individuals’ willingness to offer their vehicles for designated driver duty. If or when there is forewarning of sickness, the designated drivers will try to take quick and suitable action lest they be forced to clean up the pungent mess later in the vehicle. However, this “suitable action” might increase the risk of a crash.

### 3.5. Styles of Designated Driver

The interaction between designated driver and drunken passengers appears to differ according to how the designated driver is selected. The group may select the designated driver (or an individual may self-selects him/herself) prior to the drinking occasion (pre-selection). This person may or may not consume alcohol. The other main alternative is for the group to choose a designated driver during or after the drinking event, meaning that the candidate will likely have already consumed alcohol and may be legally impaired. This person may be the “least drunk”, although this varies.

#### 3.5.1. Pre-Selected Designated Driver (A Priori Selection)

When a peer member is pre-selected as a designated driver (that is, selected to drive prior to the social event), the role of designated driver is context specific, that is, throughout the evening, the passengers regard the selected designated driver as a friend removed from the drinking group. The role is not enduring and changes next time; however, for that period of time, there is generally tolerance for the designated driver not being “fun”.

When that happens we just let her be. She’s boring but we don’t pay much attention to her. She’ll be okay the next we go out and somebody else drives. (Female, Aged 21)

We usually do it right before we go out or earlier that day. Then we spend the night laughing at her. Man do we ever laugh. Then it’s somebody else’s turn (Male, Aged 19)

Usually if someone is chosen as a designated driver, they are stuck in that position until the next day… (Female, Aged 25)

Throughout the focus groups, participants considered the designated driver as an unselfish act, giving up pleasure for the evening and taking on the responsibility for the good of the group. But interviews with designated drivers reveal that they do not relish their role. They are more likely to consider the role negatively, and agree to it in response to the group’s needs. There is often a driver turn-taking system whereby each member is selected or self-selects to take on the role on a particular day because “it is their turn to drive”.

You are not drinking you know you aren’t having as much fun as everybody else so you don’t really want to be there while everyone else is getting smashed and you stay sober and drive. (Male, Aged 21)

Others suggested that becoming the pre-selected designated driver is borne out of their desire to “*not have a drink on the night”* or they “*got no cash for shots*” to participate as an alcohol consumer. Despite their natural dislike for the role, still they chose to take it on:

You’re not drinking and you just want to go home and you want to go to bed and you’re the designated driver and you’ve waited so long for them at the bar and you finally finish up. (Female, Aged 20)

Moreover, the positive feelings toward the designated driver do not necessarily translate into better behavior in the vehicle. In fact, the use of a pre-selected designated driver introduces an interesting paradox. Intended to be a way of increasing road safety, there was a consistent message from focus group participants that when a designated driver is pre-selected, the drinking members of the group actually drink more and feel freer to engage in inebriated, unruly pranks in the vehicle.

I know, that for me, having a designated driver gives me the green light to give her and leave the driving to somebody who can (Male, Aged 28)

When we go out we always try to have a designated driver before we leave. We can drink stupid but the driver won’t (Male, Aged 23)

As long as we have a designated driver, it’s ok and it’s acceptable to get really rip-roaring drunk (Male, Aged 22)

Pre-selected designated drivers who maintained their sobriety commented that they found their drunken passenger behaviors to be, at best, annoying and, at worst, frustrating and disturbing. They recognized the risks before they took the role and many “*just put up*” with the rowdy behavior. They expect the worst, and the worst often happens:

Getting really pissed and doing silly things with everyone wherever we go goes hand in hand (Male, Aged 23)

Sometimes, not all the time, we get bombed and laugh hysterically. We just can’t stop as we roll around on the car floor (Male, Aged 20)

#### 3.5.2. During and Post-Selected Designated Drivers (Post-Hoc Selection)

In our data, it soon became evident “designated drivers” were chosen during or after the drinking event because they were “*best able to drive*” despite having consumed various volumes of alcohol. They are likely to be the one who had the “*least to drink*”, the “*soberest*” or the last person “*still standing*”:

There’s no planning, no plan at all. You just do it on a whim sometime in the night. Not good. (Female, Aged 21)

I don’t think there is any specific part of the night where you pick one it just happens at the end of the night. Whoever is still standing! (Male, Aged 25)

*At the* end *of the night it’s decided…who is the soberest… (Male, Aged 25)*

I guess there’s been a couple of times where it’s like who’s had the least to drink. But you know, like they’re not drunk but, who’s had the least to drink. As designated as it can be. (Female, Aged 26)

It all comes down to just getting by with a little help of my friends…We have a good time (Male, Aged 21)

The later chosen “designated drivers” have already consumed drinks with the group and they are, for all practical matters, part of the drunken group. Hence, when they drive they are less inclined to try to maintain any sense of order in the vehicle and they are more likely to become active participants in the drunken passengers’ rowdy behavior:

I think it’s a hoot when they hoot and holler and fight in the back. It’s all fun. It’s a hoot and I’m all about fun even when I drive. (Male, Aged 25)

Conversely, others explained that when they are arbitrarily chosen to drive after having consumed numerous drinks, their drunken passengers, although keen to raise havoc, were more inclined to encourage and support their drunken designated driver. Sometimes this has occurred when the pre selected designated driver became too drunk or refused to drive:

Yeah, friends would be, like man, if you’re going to drink, drink, drink’ and then the next thing you know you have like five beers and your just like ooh now a new driver (Female, Aged 19)

A new driver has to be chosen, one who is more sober than the original designated driver, or one who volunteers to take over the role the original designated driver vacated. When this situation happened the drunken passengers were more apt to sublimate their desire to party in the car to the need to have a driver and to facilitate the drunken driver’s attention to the traffic:

It is a situation where, I went out with my friends, we agreed that so and so was going to be a designated driver. They all got drinking, they drank more than me. I had no choice but to drive. They could have really created a hassle for me, but they were okay in the back (Female, Aged 26)

*We’re* all *friends we gotta make sure we don’t get into trouble. We want to have fun but we don’t want to make the driver angry and then it’s not that much of a problem convincing him to continue driving. (Male, Aged 22)*

Still red lights, stop signs and parking lots often offer opportunities for both the drunken passengers and drunken designated drivers to become involved in street scuffles that are in progress. They may jump out of the vehicle and start fights with strangers or join fights that are already happening. They may become involved in what they called “fire drills” where, at a red light, all passengers and the driver jump out of the car, run around it and re-enter when the traffic light turns green. The driver usually participates or serves as a safety watch:

And then there’s the Chinese fire drill. We just have to watch that we don’t get hurt cause everyone is kind of staggering round the car (Male, Aged 22)

It’s a fire drill. It’s great fun where we all run around the car on a red – well, sometimes greet light. It depends on how it goes when someone decides to start it and everyone joins in… (Female, Aged 19)

A sober designated driver is not likely to become involved in out-of-car hooliganism and is more likely to discipline the group or take evasive action.

If there’s a fight going on outside, someone in the car is trying to start a fight with them and I just try to lock the windows on them… (Female, Aged 22)

They’re yelling out the window at people right. You can’t stop the car because these people are going to come to your car and probably rip my friends out of the vehicle. You have to avoid stopping and I think that probably becomes a danger to pedestrians as well because you’re making sudden right turns. (Female, Aged 24)

In a minority of cases, the opposite view was held, and some young people said that drunken passengers are *more* likely to make physical and psychological demands on drunken designated drivers. If, for example, the drunken designated driver tries to take control of a situation he or she disagrees with, the passengers may overrule the driver, potentially releasing the driver from their driving responsibilities.

I told them to shut up because I can’t concentrate. They were screaming at the tops of their lungs. So they forced me to drive back to the bar and they drank some more. They found a couple of buddies who then drove them back. I was kind of let go. (Male, Aged 21)

Being released from designated driver duty may also happen with a pre-selected driver who stays sober. One self-proclaimed drunken passenger proposed a kind of drunken passenger “bill of rights” that illustrates a typical drunk-passenger/designated-driver relationship. According to her, drunken passengers have the right to obnoxious behavior, of which designated driver s should not deprive them. If designated drivers take a stance for in-car order, the drunken passengers respond with a kind of moral indignation, releasing a designated driver from future assignments:

I have a group of friends where after we’re drinking the (designated driver) will drive around town singing at the top of our lungs listening to 80’s music and stuff. That’s always one of the highlights of the evening. So I mean if they don’t like it they can say no to being the (designated driver) next time… (Female, Aged 20)

## 4. Discussion

Our study of 12 focus groups conducted with young people in Alberta, Canada demonstrates that when highly intoxicated young people are driven by a designated driver who is a peer, they are likely to behave in ways that are unsafe for both driver and passengers. The role of designated driver is a difficult one. When a young person is selected from his or her peer group to be a designated driver, it often involves a tension between two roles: the temporary role of safe driver, responsible for ensuring the safety of his or her passengers; and the more enduring role of peer and friend. The demands of these two roles, friendship, and safe, responsible driving, may be incompatible. Friendship with peers implies mutuality and camaraderie, sharing activities (including partying, for many groups of young adults), and is also characterized by high degrees of peer influence, especially in adolescence and young adulthood. In contrast, the role of “designated driver” involves not participating in the partying, taking responsibility for, and taking control of, the vehicle and its passengers – often in the face of peer pressure. Whereas a sober, or even slightly intoxicated passenger is more likely to accept the driver’s temporary role as safe guarder of traffic rules and road safety, the inebriated passenger may not have sufficient judgment to do so. This may cause role conflict, not only for the designated driver (who wants to be a “buddy” as well as a safe driver), but for the inebriated passengers – who want to have fun with their friend. The ensuing social disruption has personal and interactional consequences no matter how it is played out.

From an internal, psychological point of view, this may lead to the designated driver experiencing cognitive dissonance [21]. Cognitive dissonance involves holding two conflicting ideas – the idea that it is important to let friends have this kind of fun is dissonant with the idea that such behavior is dangerous. Cognitive dissonance is unpleasant, and a key feature of the theory is that people experiencing it are motivated to reduce this dissonance by either changing or rationalizing their beliefs, attitudes or behaviors [21]. Thus, if friendship and egalitarian “peer” relationship becomes the dominant feature, the driver may rationalize the passengers’ actions as safe or even funny - spurious, playful behaviors that call for reciprocal playful manners. Alternatively, if personal responsibility of safe driving becomes the dominant belief of the designated driver, then the driver takes on a more serious role in maintaining control over the vehicle and over the passengers’ risky behavior. How these cognitive tensions are played out and their outcomes are partially dependent on the driver’s own beliefs and attitudes. However, pre-existing and emerging social interactions also have a part to play in this social arena, and these can be crucially important in whether the designated driver can ensure the safe transportation of his or her friends.

When the designated driver is selected ahead of time and remains sober (or relatively so), that person is thrust into a kind of insider/outsider, that is, he or she is a peer but is seen as being distinct on that occasion because of not participating in the drinking festivities, and because of having a different in-vehicle role than the rowdy inebriated passengers. This may lead to respect for having volunteered to take on the job of designated driver, thereby allowing the others to party without worry; however, it may also lead to him or her becoming an identified target for “pranks” or worse behavior. As an insider/outsider, the pre-selected designated driver generally attempts, for the sake of safety, to control the rowdy passengers through reason, cajoling, sanction, demands for orderly behavior or threats to stop driving, but these efforts are not always successful. The added problem of trying to prevent a drunken passenger from vomiting in the designated driver’s vehicle adds to the difficulty in safe driving. The quality of driving by a designated driver is highly dependent on his or her passengers’ behaviors, and the passengers’ actions can yield dangerous driving conditions. The designated drivers need not participate directly in the drunken passengers’ activities but s/he cannot escape the field of experience in which the high-risk events occur and influence their safety. The dilemma described by designated drivers in this study supports and explains previous findings that the designated driver role is difficult and unpopular [22]. The in-car distractions initiated by drunken passengers and negotiated by designated drivers – proceedings that occur regularly and without stigma or fanfare - can be dangerous.

Our study also confirmed the notion that designated drivers are frequently chosen during or after the social event – in that case, the driver has been drinking with his or her peers. The later-chosen designated driver is more likely to be seen (and to see themselves) as an “insider” (a contemporary, “*buddy*” or “*good friend*” who “*takes one for the gang*”) than an “insider/outsider”. This leads to less distress, cognitive dissonance and role conflict for the driver. Unfortunately, the ideal of safe driving that is assumed in the pre-select of a designated driver is absent, and selection of this kind of designated driver is almost entirely a matter of immediate practicality.

The finding that many of the in-car events, which although potentially dangerous, are sanctioned as normative practices by drunken passengers and in many cases by the designated drivers, should raise the interest of injury prevention specialists who attempt to prevent impaired driving. This research demonstrates the utmost importance of the dynamic interplay between drunken passengers and even sober designated drivers.

Perhaps the entire designated driver enterprise needs to be called into question, a formidable job because the designated driver is a popular intervention strategy that has cachet with the American and Canadian public. Hence a more insightful attempt might be considered, whereby information on how to select and behave with a designated driver should be made available to young people when they receive their driver’s license. In addition, we suggest that health promotion professionals working at the high school level to prevent impaired driving should teach young people the skills needed to become an effective designated driver, namely how to avoid in car problems with drunken passengers and how to negotiate such problems if or when they arise. It is obvious that meeting these goals is difficult and time consuming. However, people need to embrace the idea that the role of designated driver is one of safety and not “popularity and fun”. Finally, these findings suggest that future studies of designated drivers should attend to the possibility that the designated driver might be, in fact, inebriated; and to consider the annoying, stressful and potentially dangerous activities engaged in by the passengers in the vehicle.

## 5. Conclusions

The designated driver program is a popular initiative to attempt to decrease impaired driving rates and increase traffic safety. Unfortunately, in practice there are some weaknesses in this strategy, especially when young designated drivers are driving drunken peers. We report findings from a qualitative study using focus groups of 18–29 year olds and outline some of the difficulties that young designated drivers face when their passengers are very intoxicated. These difficulties range from having to deal with passengers engaging in potentially dangerous “playful pranks” to facing overtly aggressive behavior on the part of the passengers. Another hazard of being a designated driver is dealing with drunken passengers with nausea and vomiting, since attempting to avoid in-car vomiting can lead to risky driving behavior. Finally, we discuss the issue of choosing a designated driver as the “least drunk” individual in the group. The difficulties faced by young designated drivers can be viewed from the perspective of cognitive dissonance and social role conflict.

## Figures and Tables

**Table 1.** Designated driver study interview topics and examples of questions.

Topic	Examples of questions
Designated driver decision-making	When you go out with a group of friends, is there ever a designated driver? How does the group decide on who will be that driver? How is the designated driver chosen? When is a designated driver likely not to be chosen?
Designated driver in action	When someone is chosen to be the designated driver, are there pressures on him/her to have a few drinks? Are there social pressures that a designated driver, who is a friend, must endure to operate a vehicle full of drunken friends? What are they? Can you provide some examples? How do designated drivers overcome in-car pressures to socialize, yet drive? Are there times you would prefer not to use a designated driver when you go drinking?
